# Mesenteric Venous Malformation Case Report in an Adolescent With Midgut Volvulus

**DOI:** 10.1155/cris/2730309

**Published:** 2026-01-16

**Authors:** Yeu Sanz Wu, Philip J. Katzman, Suzie A. Noronha, Nicole A. Wilson

**Affiliations:** ^1^ Department of Surgery, University of Rochester Medical Center, 601 Elmwood Avenue, Rochester, New York, USA, rochester.edu; ^2^ Departments of Pathology and Laboratory Medicine, University of Rochester Medical Center, Rochester, New York, USA, rochester.edu; ^3^ Division of Pediatric Hematology and Oncology, Department of Pediatrics, University of Rochester Medical Center, 601 Elmwood Avenue, Rochester, New York, USA, rochester.edu; ^4^ Division of Pediatric Surgery, Department of Surgery, University of Rochester Medical Center, 601 Elmwood Avenue, Rochester, New York, USA, rochester.edu; ^5^ Department of Biomedical Engineering, University of Rochester, 601 Elmwood Avenue, Rochester, New York, USA, rochester.edu; ^6^ Section of Pediatric Surgery, Department of Surgery, Oklahoma Children’s Hospital, University of Oklahoma Health Campus, 1200 Everett Drive, Oklahoma City, Oklahoma, USA, ou.edu

**Keywords:** case report, congenital vascular malformation, hemangioma, intra-abdominal cyst, mesenteric venous malformation, volvulus

## Abstract

**Introduction:**

Although the reported incidence of congenital vascular malformations is ~1.5% of the general population, the true incidence of these lesions is difficult to assess due to the heterogeneity of vascular anomalies and the variability in terminology used in reporting. These vascular anomalies can involve capillaries, lymphatics, venous, and/or arterial structures and can occur anywhere in the body. Rarely does a vascular malformation originate from the gastrointestinal (GI) mesentery and present as a bowel obstruction.

**Case Report:**

This report describes an adolescent patient with an unusual presentation of a vascular malformation involving the GI mesentery, manifesting as midgut volvulus. Emergent laparotomy revealed a large intra‐abdominal cystic structure that volvulized resulting in a small bowel obstruction. The lesion and involved segment of small bowel were resected and found to be a mesenteric venous malformation on pathology.

**Conclusion:**

Vascular anomalies of the GI tract are uncommon but should be included in the broad differential for patients presenting with abdominal pain, symptoms consistent with a small bowel obstruction, and/or a cystic intra‐abdominal mass. In addition, utilization of accurate and standardized terminology when reporting these lesions is important to facilitate prompt and accurate diagnosis and treatment of patients and to establish a reliable foundation of continued research on vascular anomalies.

## 1. Introduction

Vascular anomalies represent a broad array of neoplastic and non‐neoplastic entities. Mulliken and Glowacki’s [1] classification, proposed in 1982, was based on endothelial characteristics of the lesion, and this was further formalized by the International Society for the Study of Vascular Anomalies (ISSVA) in 2018 [2]. Vascular anomalies were categorized as either a *tumor*, if it involves a proliferative component or endothelial cell hyperplasia, or a *malformation*, if it has normal endothelial cell turnover [3].

The proposed terminology has not been strictly followed, leading to confusion about the exact entities being reported [2]. Up to 71.3% of hemangioma cases may be misclassified [4], with the term “hemangioma” being previously used to describe both vascular tumors and malformations [2, 5]. This can lead to suboptimal or incorrect treatment for these conditions [2, 6]. Other misnomers have also persisted. Entities historically called “cavernous hemangiomas” most often represent morphological abnormalities of veins [5] and, therefore, are more appropriately named venous malformations.

Overall, the reported incidence of congenital vascular malformations is 1.5% [7]. They may involve capillaries, lymphatics, venous, and/or arterial structures and can occur anywhere in the body. Vascular malformations of the gastrointestinal (GI) tract are uncommon, and those of the GI mesentery are even more rare [8, 9]. However, the exact incidence of mesenteric malformations is difficult to estimate, likely due to inconsistent terminology. Prior reports of “cavernous hemangiomas” of the small intestine are mostly in adults who present with GI bleeding or anemia due to involvement of the bowel wall [10]. Yang et al. [8] reported one of the first pediatric cases of a giant, mixed cavernous and venous hemangioma arising from the small intestine mesentery. Other pediatric cases of small intestine mesenteric hemangiomas have been reported, but it is unclear whether these represent true hemangiomas (tumors) or venous malformations [11].

The purpose of this report is to describe an unusual case of a 13‐year‐old female with a large venous malformation located in the small intestine mesentery. We also review the available cases of GI mesenteric “hemangiomas” in the pediatric population with the goal of differentiating hemangiomas from venous malformations.

## 2. Case Presentation

This case is reported following the CARE guidelines (Suppporting Information 1). A 13‐year‐old female with no significant past medical history presented to the emergency department (ED) with intractable vomiting. She reported a 1‐week history of nausea, abdominal pain, and emesis that progressed to bilious, eventually becoming intractable with an inability to tolerate any oral intake, including liquids. In the ED, she was afebrile, tachycardic to 134 bpm, and mildly hypotensive (100/61 mmHg). On exam, she had mild, diffuse abdominal tenderness without evidence of peritonitis. Laboratory investigation demonstrated moderate electrolyte derangements, with chloride 89 mmol/L, anion gap 26, blood urea nitrogen 41 mg/dL, and acute kidney injury, with creatinine of 1.35 mg/dL. She also had evidence of hemoconcentration with a white blood cell count of 11.1 1000/µL, hemoglobin 15.6 g/dL, and hematocrit 45%. A computed tomography (CT) scan of her abdomen and pelvis was performed. Intravenous contrast was not utilized due to the acute kidney injury and no oral contrast was given due to her nausea with intractable vomiting and, thus, concern for aspiration. The CT scan demonstrated a large cystic structure obscuring much of the intra‐abdominal cavity (Figure 1A) and mesenteric swirling (Figure 1B). The cyst appeared simple and thin‐walled in nature without loculations. Due to concern for volvulus causing a closed‐loop bowel obstruction, she was promptly brought to the operating room for exploratory laparotomy.

Figure 1(A) Large cystic structure obscuring much of the intraabdominal cavity on coronal view with dilated loops of small bowel. (B) Noted characteristic swirling pattern on axial view (white arrow).

**Figure:**
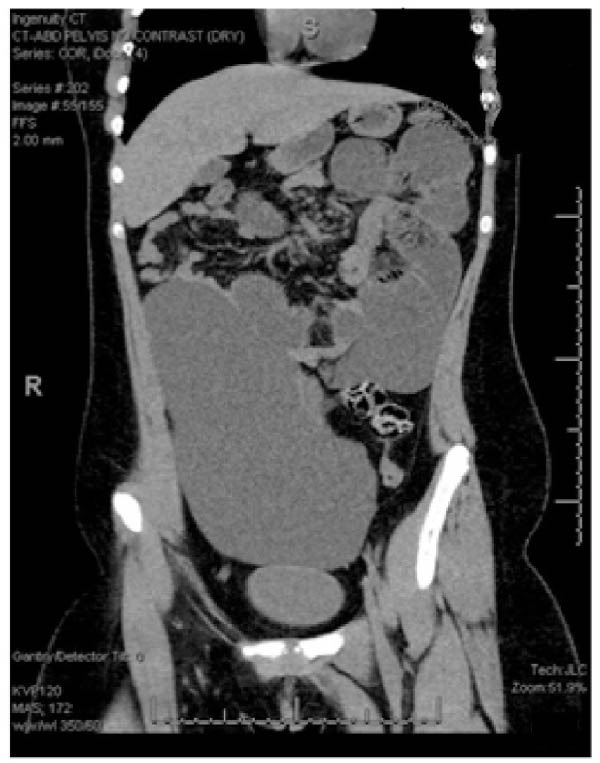
(A)

**Figure:**
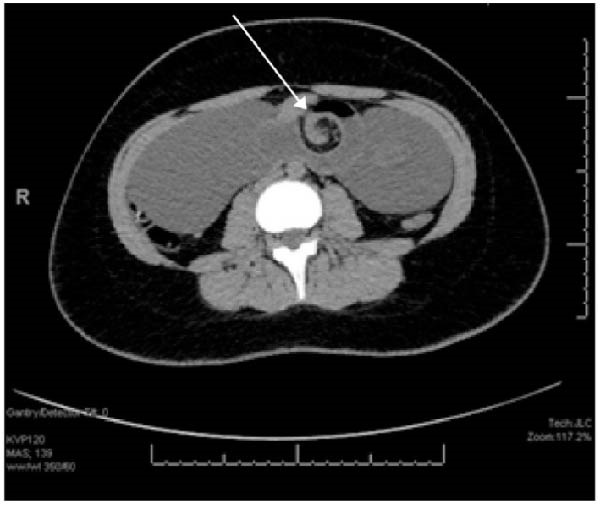
(B)

**Figure 2:**
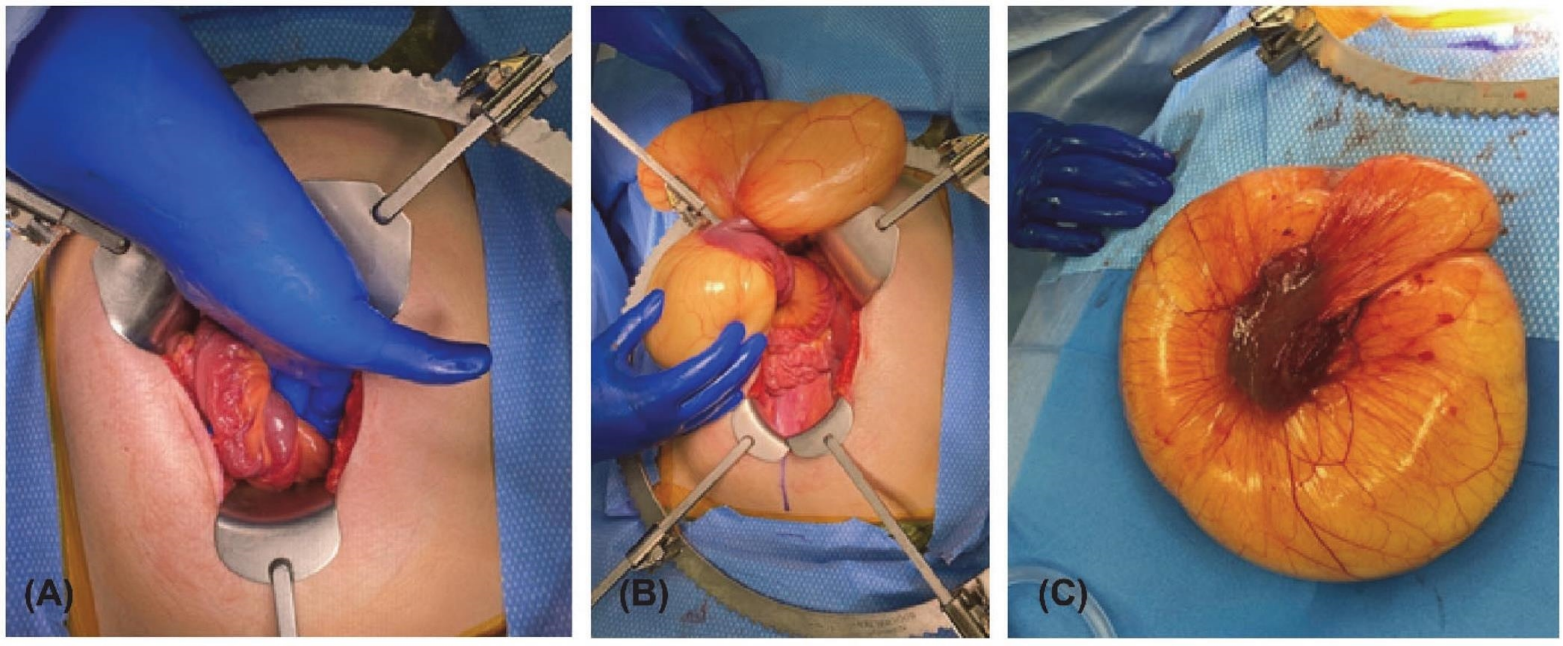
Intraoperative photos. (A) Intially noted volvulized bowel with approximately two twists. (B) Large, fluid‐filled cystic mass with dumbell‐like shape. The cyst was intimately involved with a short segment of small intestine. (C) 18.5 cm × 9.5 cm × 2 cm cystic mass resected en bloc, intact with a segment of small bowel. The cyst degenerated in specimen container and the cyst fluid was noted to be tan and cloudy in appearance.

Upon entry into the abdomen through an upper midline incision, a large, fluid‐filled cyst‐like structure was encountered. The cystic structure was indistinguishable from the small bowel mesentery and was volvulized around and intimately associated with a short segment of small intestine (Figure 2A,B). The large cystic structure, which measured 18.5 cm × 9.5 cm × 2 cm (Figure 2C), was carefully externalized from the abdomen. The cystic mass and the associated 9‐cm portion of the small intestine were resected en bloc without disruption of the cyst (Figure 2C). A primary side‐to‐side, functional end‐to‐end, small bowel anastomosis was created. The rest of the small and large intestines were examined, and no additional abnormalities were noted. The patient tolerated the procedure well and progressed uneventfully through her postoperative course and was discharged home on postoperative Day 5. She was seen in follow‐up approximately 1 month later and was tolerating a regular diet without abdominal pain and was returned to full, unrestricted activity.

**Figure 3:**
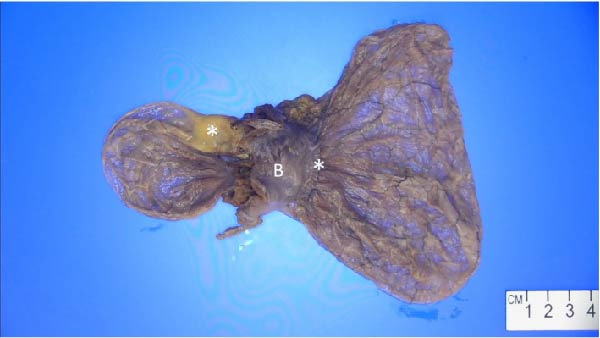
The thin‐walled cyst (18.5 cm × 9.5 cm × 2 cm), which contained ~1200 mL of a tan cloudy fluid, attaches to the mesentery (asterisk) of the small bowel (B) and does not communicate with the bowel lumen. The cyst lining, shown here, is smooth and contains a fine network of small vessels. Photo by: Elizabeth Sharratt, PA (ASCP) CM.

**Figure 5:**
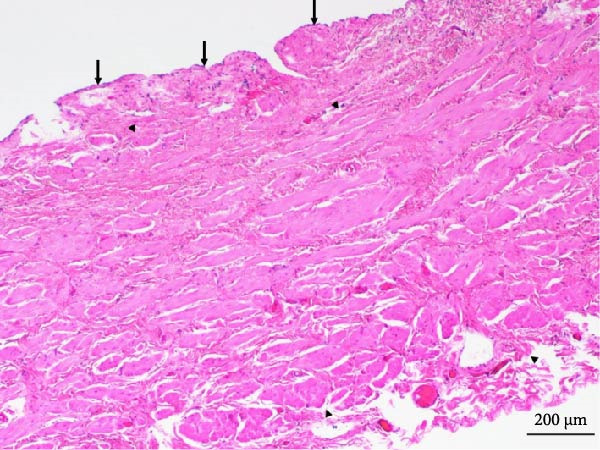
This part of the cyst wall is thicker and contains a broader array of smooth muscle fibers (between arrowheads) and is lined by an endothelium (arrows), consistent with a large vessel wall (original magnification: 40x).

After an extensive and thorough pathologic analysis, the specimen was noted to contain a mesenteric venous malformation. Initial gross examination demonstrated a thin‐walled cyst that contained ~1200 mL of a tan cloudy fluid, was attached to the mesentery (Figure 3, asterisk) of the small bowel (Figure 3B), and did not communicate with the bowel lumen (Figure 3). The cyst lining was smooth and contained a fine network of small vessels. The endothelial lining of the cyst wall was positive for CD31 vascular immunostain (Figure 4) and small vessels in the cyst wall were also positive for CD31. A cystic lymphatic malformation was excluded due to a negative D2−40 immunostain. D2−40 and CD31 are useful immunohistochemical markers for vascular lesions. While CD31 is a general vascular marker that stains the endothelium of both blood vessels and lymphatics, D2−40 is a more specific marker for lymphatic endothelium [12]. Mesothelial markers (calretinin and WT‐1 immunostains) were also negative. Some of the small vessels were positive for D2−40 immunostain (Figure 4), consistent with small lymphatics. One portion of the cyst wall was thicker, contained a broader array of smooth muscle fibers (Figure 5), and was lined by an endothelium, consistent with a large vessel wall.

Figure 4(A) The cyst wall has an endothelial lining (arrows in A and B) and (B) a variable number of smooth muscle fibers (arrowheads), small vessels (V), and scattered adipose tissue (fat, F). (C) The endothelial lining is positive for CD31 vascular immunostain (arrows) and small vessels in the wall (arrowheads) are also positive. (D) The endothelium is negative for the lymphatic marker, D2‐40, but some of the small vessels are positive for this immunostain (arrowheads), consistent with small lymphatics (original magnifications: A, C, D–40x; B–10x).

**Figure:**
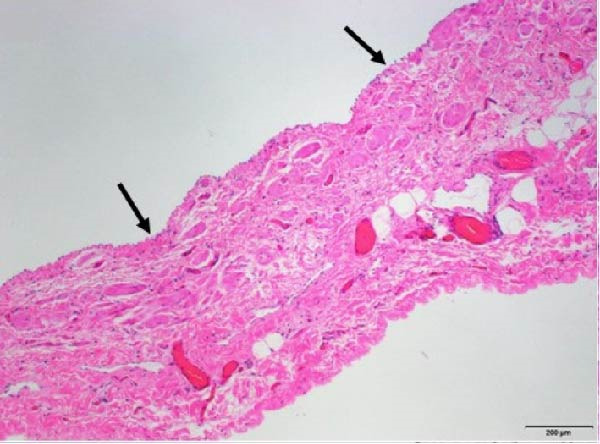
(A)

**Figure:**
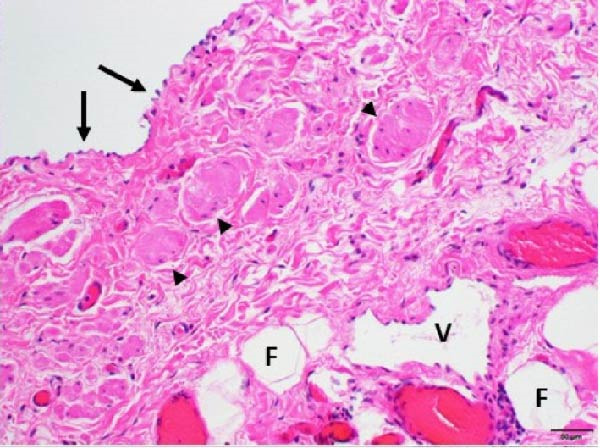
(B)

**Figure:**
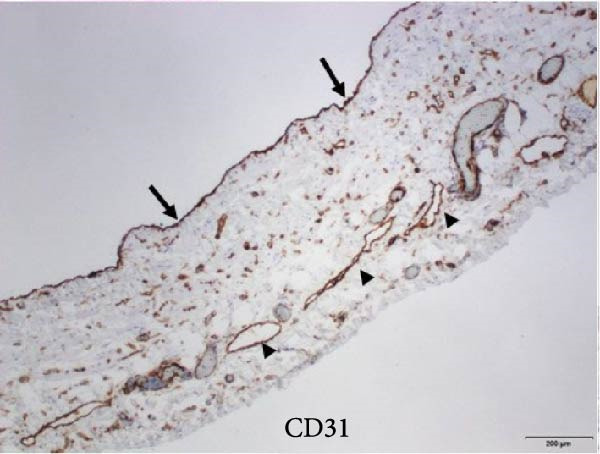
(C)

**Figure:**
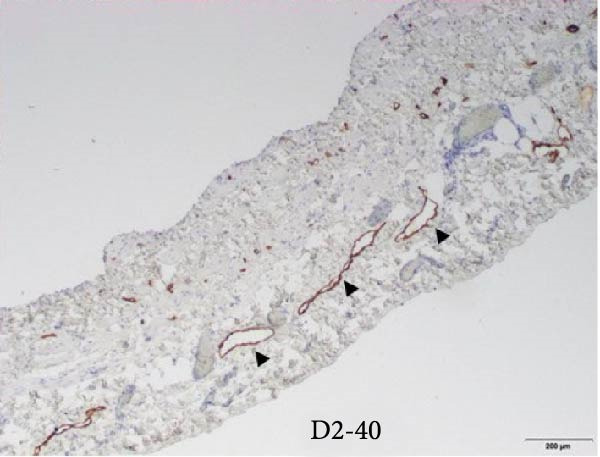
(D)

## 3. Discussion

Vascular malformations are anomalies that occur during morphogenesis of the embryonic vascular system between the 4th and 10th weeks of intrauterine life [13, 14]. They can be subdivided into high‐flow lesions (e.g., arteriovenous malformation) and low‐flow lesions, which are of capillary, venous, lymphatic, or hybrid origin. This case represents a rare incidence of midgut volvulus due to a low‐flow, macrocystic, *mesenteric venous malformation* in the small intestine mesentery of a pediatric patient.

Venous malformations are typically characterized by a soft, compressible, nonpulsatile mass [13]. Characteristic histopathologic findings of venous malformations include ectatic, thin‐walled abnormal vessels [8] in which smooth‐muscle actin (SMA) staining reveals muscle in clumps instead of normal smooth muscular architecture [2]. The reported incidence of venous malformations is 1–2 per 10,000 births and affects males and females in a 1:1 ratio [13, 14]. These congenital lesions are not always apparent at birth but typically grow in proportion to the somatic growth of the child [14]. Bleeding, inflammation, or hormonal changes can result in sudden enlargement [13, 15]. In this reported case, the cystic component enlarged to the point where it became a nidus for consequent volvulus of the associated small intestine, leading to a small bowel obstruction.

Although uncommon, there are numerous reports of enteric hemangiomas. They account for 7%–10% of all benign tumors of the small intestine and are most commonly found in the jejunum [16, 17]. In contrast to mesenteric vascular malformations, which are isolated to the mesentery without involving the bowel wall, enteric hemangiomas are vascular tumors that typically involve the bowel wall and cause GI bleeding or anemia [18]. The mean age of initial bleeding symptoms is reported at 5 years; however, given the difficulty with accurate diagnosis and nonspecific presentation, age of definitive diagnosis ranges from 2 months to 79 years [19]. The older age range for time of definitive diagnosis suggests that not all enteric hemangiomas spontaneously involute.

The true incidence of *GI mesenteric* venous malformations is unknown. In Table 1, we provide details from all the available case reports of GI mesenteric venous malformations in the pediatric population and attempt to differentiate venous malformations from hemangiomas based on reported histopathologic characteristics (Table 1) [20–24]. We were able to identify fewer than five previously reported pediatric cases in the English‐language literature (Table 1).

**Table 1:** Available case reports of GI mesenteric venous malformations in the pediatric population.

Case reports	Age	Presenting symptoms/location	Gross	Micro	D2‐40^a^	CD31^b^	SMA^c^	Probable diagnosis
Wu et al. (current case)	13 year female	• Abdominal pain and vomiting • Small bowel obstruction • Jejunum mesentery	• Thin‐walled cyst (18.5 cm × 9.5 cm × 2 cm) • Cyst lining contains fine network of small vessels • Does not communicate with bowel lumen	• Endothelial lining • Variable number of smooth muscle fibers, small vessels, and scattered adipose tissue	Negative	Positive	Not available	Cystic venous malformation

Komura et al. [20]	9 year female	• Abdominal pain and constipation • Sigmoid colon mesentery	• Lobulated, septated, cystic mass (11.5 × 7.2 × 5.6 cm) • Red coloration • Adjacent bowel was not involved	• Simple squamous endothelial lining	Negative	Positive	Not available	Cystic venous malformation

Yang et al. [8]	5 year female	• Abdominal pain and vomiting • Small intestine mesentery	• Honeycomb mass (16 × 8 × 5 cm) • Neoplasm located in mesentery • Bowel wall not involved, but hemangioma adherent to bowel wall	• Diluted thin‐walled vessels (majority) • Thick‐walled vessels with less organized smooth muscle (minority)	Negative	Positive	Positive	Mixed venous malformation and venous hemangioma

Thambidorai et al. [11]	4 year female	• Abdominal pain, vomiting, and fever	• At laparotomy, described as ‘firm mass’ (8 × 5 × 5 cm) • Predominantly thin‐walled vessels with slow filling on compression • Adjacent bowel was not involved	• Numerous flattened endothelium‐lined vascular spaces, varying sizes, areas of focal thrombosis • Scattered lymphatic follicles with germinal centers within vessel walls and surrounding stroma	Not available	Not available	Not available	Low‐flow vascular malformation (less likely) vs. Involuting hemangioma (more likely)

^a^The D2−40 immunostain is a common lymphatic marker [20]. Negative D2−40 may help rule out a cystic lymphatic malformation as the diagnosis up to a certain size [21].

^b^CD31, also known as platelet endothelial cell adhesion molecule 1 (PECAM‐1), is a marker for vascular differentiation [22]. Positive CD31 supports a vascular origin.

^c^SMA (Smooth Muscle Actin) stains pericytes around normal vessels and benign vascular proliferations [23]. SMA positivity further supports a vascular origin.

Alternative management options for venous malformations include percutaneous sclerotherapy using a fibrosing material (e.g. sodium tetradecyl sulfate, alcohol, an alcoholic solution of zein [i.e., Ethibloc]) [13, 14]. However, in the pediatric population, mesenteric venous malformations may represent a special case. Sclerotherapy applied to the mesentery carries the risk of compromising the vascular supply to the intestine and may result in intestinal ischemia, necrosis, and/or perforation [23, 25]. Surgical resection is indicated in cases in which an oncologic process cannot be excluded or if an extensive bowel resection is not required to remove the isolated mesenteric venous malformation [23]. Percutaneous sclerotherapy prior to surgical resection may help limit bleeding and other complications [13]. In this case, the preoperative diagnosis was volvulus causing a bowel obstruction without prior knowledge of an existing mesenteric venous malformation, thus did not allow for precursory percutaneous sclerotherapy.

## 4. Conclusion

Although vascular anomalies of the GI tract are uncommon, they should be included in the broad differential for patients presenting with abdominal pain, small bowel obstruction, and/or a cystic intra‐abdominal mass. This report underscores the importance of using the correct terminology when discussing vascular malformations. Due to the complicated classification, tendency for use of imprecise terminology, and need for multimodal therapy, pediatric patients with vascular malformations benefit from a multidisciplinary team approach that includes pediatric specialists in radiology, hematology, dermatology, plastic surgery, vascular surgery, otolaryngology, pathology, and general surgery [26].

## Conflicts of Interest

The authors declare no conflicts of interest.

## Funding

No funding was secured for this study.

## Supporting Information

Additional supporting information can be found online in the Supporting Information section.

## Supporting information


**Supporting Information** Completed Case Report (CARE) guidelines.

## Data Availability

The data that support the findings of this study are available from the corresponding author upon reasonable request.
